# *LRP6* Polymorphisms Is Associated With Sudden Cardiac Death in Patients With Chronic Heart Failure in the Chinese Han Population

**DOI:** 10.3389/fcvm.2021.815595

**Published:** 2022-02-04

**Authors:** Qi Guo, Yiwei Lai, Jianmin Chu, Xuhua Chen, Mingyang Gao, Caihua Sang, Jianzeng Dong, Jielin Pu, Changsheng Ma

**Affiliations:** ^1^Department of Cardiology, National Clinical Research Center for Cardiovascular Diseases, Beijing Anzhen Hospital, Capital Medical University, Beijing, China; ^2^State Key Laboratory of Cardiovascular Disease, National Center for Cardiovascular Disease, Fuwai Hospital, Chinese Academy of Medical Sciences and Peking Union Medical College, Beijing, China; ^3^Department of Cardiology, Shanghai East Hospital, Tongji University, Shanghai, China

**Keywords:** LRP6, single-nucleotide polymorphism, chronic heart failure, prognosis, sudden cardiac death

## Abstract

Low-density lipoprotein receptor-related protein 6 (LRP6) plays a critical role in cardiovascular homeostasis. The deficiency of *LRP6* is associated with a high risk of arrhythmias. However, the association between genetic variations of *LRP6* and sudden cardiac death (SCD) remains unknown. This study aims to explore the association between common variants of *LRP6* and the prognosis of chronic heart failure (CHF) patients. From July 2005 to December 2009, patients with CHF were enrolled from 10 hospitals in China. The single-nucleotide polymorphism (SNP) rs2302684 was selected for the evaluation of the effect of *LRP6* polymorphisms on the survival in patients with CHF. A total of 1,437 patients with CHF were finally included for the analysis. During a median follow-up of 61 months (range 0.4–129 months), a total of 546 (38.0%) patients died, including 201 (36.8%) cases with SCD and 345 (63.2%) cases with non-SCD. Patients carrying A allele of rs2302684 had an increased risk of all-cause death (adjusted HR 1.452, 95% CI 1.189–1.706; *P* < 0.001) and SCD (adjusted HR 1.783, 95% CI 1.337–2.378; *P* < 0.001). Therefore, the SNP rs2302684 T>A in *LRP6* indicated higher risks of all-cause death and SCD in patients with CHF. *LRP6* could be added as a novel predictor of SCD and might be a potential therapeutic target in the prevention of SCD in the CHF population.

## Introduction

Chronic heart failure (CHF), which may be caused by ischemic cardiomyopathy (ICM) or non-ischemic cardiomyopathy (NICM), is one of the chief causes of morbidity and mortality worldwide (1, 2). It currently affects more than five million Americans and the prevalence is expected to increase by 25% within the next 15 years (3). This heart failure pandemic is also evident in Asia and China (4). The predominant modes of death in CHF patients are pump failure and sudden cardiac death (SCD) (5). Sudden cardiac death, caused by malignant ventricular tachycardia (VT) or ventricular fibrillation (VF), remains a primary cause of mortality in patients with CHF (6). Therefore, the prediction and prevention of SCD play critical roles in the management of the CHF population. So far, even several factors have been known as potential predictors of SCD, including biomarkers, hemodynamic status, and electrophysiological parameters, the sensitivity and specificity are not powerful (7).

Low-density lipoprotein (LDL) receptor-related protein 6 (LRP6) is a single-pass transmembrane protein, which contains four extracellular epidermal growth factor-like repeats and three LDL receptor repeats (8). It is recognized as a coreceptor for the Wnt signaling cascade and plays a critical role in regulating Wnt signaling (9, 10). Work to date has identified that dysregulated Wnt signaling conduces to a high incidence of arrhythmias associated with various forms of heart disease (11). Furthermore, accumulating evidence reveals the significant effect of LRP6 on cardiovascular health and homeostasis (12). Additionally, LRP6 was mainly allocated within the gap junction of cardiomyocytes (13). However, the potential relation between genetic variations of *LRP6* and SCD has not yet been reported in previous studies. In the present study, we examined the association between common variants of *LRP6* and the prognosis in patients with CHF.

## Materials and Methods

### Study Population

From July 2005 to December 2009, patients with CHF were enrolled from 10 hospitals in China. Details for the cohort have been described previously (9, 14–16). Inclusion criteria include: (a) CHF caused by ICM or idiopathic dilated cardiomyopathy (DCM); (b) classification of the New York Heart Association (NYHA) was II–IV with optimizing drug therapy; and (c) left ventricular ejection fraction (LVEF) ≤ 50% in ICM or ≤ 45% in DCM (9, 14–16). Ischemic cardiomyopathy was diagnosed as ≥70% luminal stenosis of one or more major coronary arteries diagnosed by coronary angiography with a myocardial infarction history at least 3 months before the enrolment. DCM was diagnosed consistently with the guidelines of familial DCM (17). Excluded criteria include: (a) pacemaker dependency; (b) unable to perform the genotyping; and (c) pregnancy, terminal illnesses, or other uncontrollable system diseases (9, 14–16).

The study was approved by the Ethics Committee of Beijing Anzhen Hospital and Fuwai Hospital (Beijing, China), and complied with the principles of the Declaration of Helsinki. Written informed consent was obtained from all the enrolled patients who reported themselves as Chinese Han nationality.

### Endpoint Evaluation

All the participants were followed up periodically until August 2017 during regular outpatient clinics or by transtelephonic visits. The endpoints included all-cause death, SCD (ICD appropriate discharge was regarded as SCD), and non-SCD (NSCD) (heart transplantation was regarded as non-SCD). Sudden cardiac death was defined as an unexpected death within 1 h of onset of acute symptoms attributable to cardiac causes or an unwitnessed death of someone last seen in a stable condition in 24 h without evidence of non-cardiac causes (7). If there were discrepancies between the first two reviewers, the event was adjudicated by a third investigator to provide the final classification.

### Tag Single-Nucleotide Polymorphisms Selection

Tag single-nucleotide polymorphisms (SNPs) were selected by the pairwise tagging method from the HapMap CHB databank (HapMap Data Rel 24 PhaseII, Nov08, on NCBI B36 assembly, dbSNP b126) via the tag SNPs' online software (http://hapmap.ncbi.nlm.nih.gov/cgi-perl/gbrowse/hapmap24_B36/#search). Common variants were defined as a minor allele frequency (MAF) > 0.05, with a linkage disequilibrium (LD) measure *r*^2^ threshold of 0.8. Forty-three tag SNPs that covered the entire *LRP6* gene were selected. To reduce the false-positive caused by multiple tests, with 43 candidate SNPs, *P* < 0.001 was considered statistically significant for SNP selection. Polymerase chain reactions (PCRs) were performed firstly in 100 subjects with CHF. There were no significant associations between SNPs and clinical endpoints other than the SNP rs2302684 T>A. Thus, rs2302684 was finally selected to perform the PCRs in the whole study population for the analysis.

### Genotyping in CHF Population

Genomic DNA was extracted from peripheral blood leukocytes of the participants and stored at −70°C after determination of absorbance at 260 nm followed by Picogreen analysis (Molecular Probes, Eugene, Oegon, USA) (18).

Primers were designed by Primer Premier 5.0 software as follows: forward TTGATGATGCTCCTGTAA and reverse TATTCTTGGCCTTGTTCT (328 bp). PCR amplification was performed with the Geneamp PCR system 9700 (Applied Biosystems). An initial 4 min cycle at 94°C was followed by 35 thermal cycles at 94°C for 30 s, 47°C for 30 s, and 72°C for 30 s, and ended with a 10 min extension at 72°C. Each reaction mixture (30 μl) contained 3 μl 10 × PCR buffer, 0.5 μl dNTP-mix (10 mmol/L), 0.5 μl of each primer (10 μmol/L), 0.5 μl Taq polymerase (5 U/μl; Takara Bio), 1 μl genomic DNA (50 ng/μl), and 24 μl double-distilled H_2_O.

**Figure 1:**
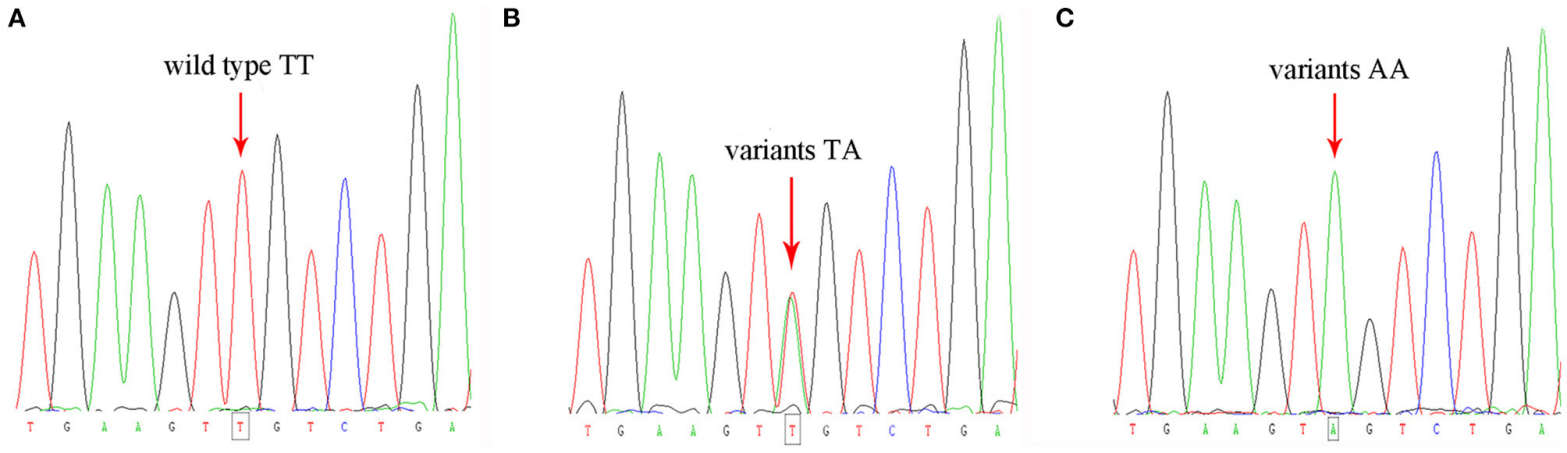
Genotyping of the studied population showing the wild type **(A)** and the variants of rs2302684 T>A **(B,C)**.

PCR products were sequenced after purification by ABI 3130XL DNA Analyzer System (Applied Biosystems, USA). Repeat genotyping was carried out in 95 (5.0%) random duplicate samples to affirm the reproducibility was 100% (Figure 1).

### Statistical Analysis

Continuous variables were presented as the mean ± SD and compared by Student's test. Categorical variables were presented as numbers and percentages and compared by chi-square analysis. A *P*-value of <0.05 was considered statistically significant. Linkage disequilibrium of rs2302684 was analyzed by Haploview4.2, and Hardy-Weinberg equilibrium of alleles was analyzed by chi-square analysis with 1 degree of freedom. Survival analysis was performed in CHF patients. Cox proportional hazards models were performed under three different models (dominant, recessive, additive models) to evaluate the effects of genotype on survival. Kaplan-Meier curve was applied to describe survival freedom from events and multivariate cox proportional hazards models were used to adjust for confounding factors. The covariates were selected according to clinical significance and baseline data, including age, sex, NYHA levels, LVEF, ischemic etiology, and other variables with a *P*-value of <0.2 in the baseline. Statistical analyses were conducted using the IBM SPSS 26.0 software.

## Results

### Clinical Characteristics of the Studied Population

A total of 1,437 patients (age 60.55 ± 11.98 years, 1,134 males) with CHF were finally enrolled for the analysis, including 957 patients with ICM and 480 patients with DCM. The mean LVDD of the participants was 63.27 ± 9.77 mm, and the mean LVEF was 36.04 ± 8.80%. Among them, 43.0% of patients had the NYHA function of level II, 32.6% of patients had the NYHA function of level III, and the other 24.4% patients had the NYHA function of level IV. The mean BMI of the participants was 24.84 ± 3.84 kg/m^2^. The clinical characteristics were summarized in Table 1.

**Table 1:** Baseline characteristics of the studied population.

**Clinical characteristics**	**CHF**			
	**ALL (*n* = 1,437)**	**TT (*n* = 994)**	**TA+AA (*n* = 443)**	***P*-values**
Sex, *n* = Male (%)	1,134 (78.9)	794 (79.9)	340 (76.7)	0.203
Age (years)	60.55 ± 11.98	60.16 ± 12.05	61.41 ± 11.79	0.068
Etiology, *n* = ICM (%)	957 (66.6)	640 (64.4)	317 (71.6)	0.009
LVDD (mm)	63.27 ± 9.77	63.15 ± 9.57	63.55 ± 10.21	0.479
LVEF (%)	36.04 ± 8.80	36.26 ± 8.54	35.55 ± 9.36	0.157
NYHA, *n* (%)				0.047
I	0	0	0	–
II	618 (43.0%)	446 (44.9)	172 (38.8)	–
III	469 (32.6%)	322 (32.4)	147 (33.2)	–
IV	350 (24.4%)	226 (22.7)	124 (28)	–
BMI (kg/m^2^)	24.84 ± 3.84	24.72 ± 3.80	25.10 ± 3.91	0.085
Hypertension, *n* (%)	723 (50.3)	493 (49.6)	230 (51.9)	0.45
Hyperlipidemia, *n* (%)	353 (24.6)	249 (25.1)	104 (23.5)	0.566
Diabetes, *n* (%)	361 (25.1)	245 (24.6)	116 (26.2)	0.579
QRS durations (ms)	110.45 ± 52.48	110.74 ± 53.36	109.80 ± 50.51	0.755
AF/AFL, *n* (%)	214 (14.9)	151 (15.2)	63 (14.2)	0.692
PVC/NSVT, *n* (%)	114 (7.9)	72 (7.2)	42 (9.5)	0.179
VT/VF, *n* (%)	43 (3.0)	27 (2.7)	16 (3.6)	0.452
Medications, *n* (%)				
β-blocker	1077 (74.9)	733 (73.7)	342 (77.2)	0.184
ACEI	992 (69.0)	686 (69.0)	309 (69.8)	0.828
Diuretic	1055 (73.4)	720 (72.4)	333 (75.2)	0.309
Aldosterone antagonists	1066 (74.2)	738 (74.2)	328 (74.0)	0.987
ICD, *n* (%)	37 (2.6)	24 (2.4)	13 (2.9)	0.693

*AF, atrial fibrillation; AFL, atrial flutter; BMI, body mass index; CHF, chronic heart failure; DCM, dilated cardiomyopathy; ICM, ischemic cardiomyopathy; LVDD, left ventricular end-diastolic diameter; LVEF, left ventricular ejection fraction; NSVT, non-sustainable ventricular tachycardia; NYHA, New York Heart Association; PVC, premature ventricular contraction; VT, ventricular tachycardia; VF, ventricular fibrillation*.

*M ± SD for normally distributed data and n (%) for categoric variables*.

In the studied CHF population, 443 patients (age 61.41 ± 11.79 years, 340 males) carried A allele of SNP rs2302684. The patients carrying A allele of SNP rs2302684 comprised a larger percentage of patients with ICM than those carrying the wild type of TT (71.6 vs. 64.4%, *P* = 0.009). Furthermore, CHF patients with A allele of rs2302684 had a tendency to the higher classification of NYHA level than those without (NYHA II, 38.8 vs. 44.9%; NYHA III, 33.2 vs. 32.4%; NYHA IV, 28 vs. 22.7%; *P* = 0.047). However, no significant differences were demonstrated in age, sex distribution, BMI, LVDD, LVEF, complications, the prevalence of arrhythmias, and medication between the patients with rs2302684 wild type of TT and those with A allele (Table 1).

### Long-Term Follow-Up of the CHF Population

During a median follow-up of 61 months (range 0.4–129 months) in 1,437 participants with CHF, a total of 546 (38.0%) patients died, including 348 patients with ICM and 198 patients with DCM. Among them, 201 (36.8%) cases had SCD, including 129 cases with ICM and 72 cases with DCM. The rest of 345 (63.2%) cases had NSCD.

### Associations of SNP Rs2302684 T>A and the Clinical Endpoints

**Figure 2:**
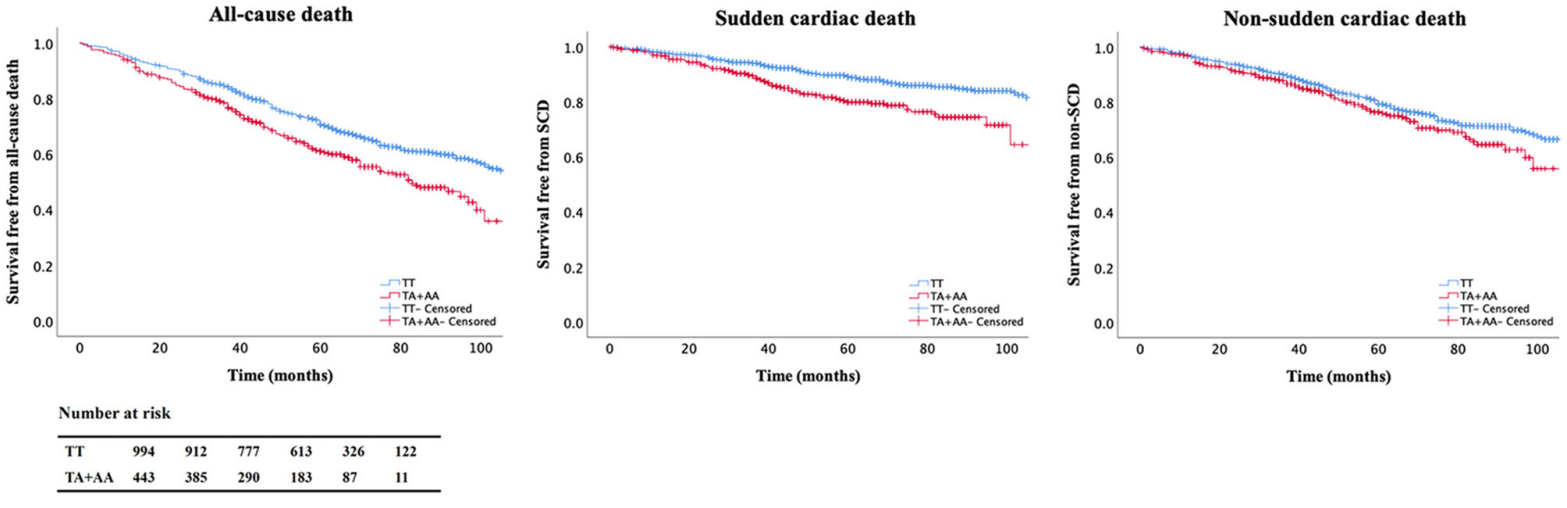
Kaplan-Meier curves in the chronic heart failure (CHF) cohort. Patients carrying A allele of rs2302684 were more vulnerable to all-cause death and sudden cardiac death (SCD) than those without it. The table denotes the number of patients at risk for every 20 months of the follow-up.

The correlation of mortality and A allele of rs2302684 were analyzed by survival cox regression analysis in the CHF cohort under the dominant, recessive, and additive models, respectively. The effect of the A allele on mortality was significant under different models (Table 2). Under the dominant model, the risks of all-cause death (HR 1.45, 95% CI 1.21–1.73; *P* < 0.001) and SCD (HR 1.85, 95% CI 1.39–2.45; *P* < 0.001) increased significantly in patients with A allele of rs2302684. After adjusted for age, sex, ischemic etiology, NYHA levels, LVEF, BMI, and the use of β-blocker, the associations remained significant in all-cause death (HR 1.43, 95% CI 1.20–1.72; *P* < 0.001) and SCD (HR 1.80, 95% CI 1.34–2.40; *P* < 0.001). The Kaplan-Meier curves made under the dominant models were shown in Figure 2. Thus, SNP rs2302684 T>A indicated a higher risk of all-cause death and SCD but not NSCD in the CHF patients.

**Table 2:** Associations of SNP rs2302684 T>A and clinical endpoints of the studied population.

**Models**	**Dominant (TA** **+** **AA compared with TT)**			**Recessive (AA compared with TT** **+** **TA)**			**Additive models**
	**TT**	**AA + TA**	**HR (95% CI)**	**TT + TA**	**AA**	**HR (95% CI)**	**HR (95% CI)**
**All cause death**	361	185		521	25		
Unadjusted			1.45 (1.21–1.73)			1.83 (1.22–2.73)	1.40 (1.21–1.63)
Adjusted^*^			1.43 (1.20–1.72)			1.87 (1.25–2.79)	1.39 (1.20–1.62)
**SCD**	121	80		189	12		
Unadjusted			1.85 (1.39–2.45)			2.36 (1.3–4.23)	1.71 (1.36–2.14)
Adjusted^*^			1.80 (1.34–2.40)			2.34 (1.33–4.29)	1.68 (1.33–2.12)
**NSCD**	240	105		332	13		
Unadjusted			1.25 (0.99–1.57)			1.51 (0.87–2.64)	1.23 (1.01–1.50)
Adjusted^*^			1.24 (0.98–1.57)			1.54 (0.88–2.69)	1.23 (1.01–1.50)

*CHF, chronic heart failure; HR: NSCD, non-sudden cardiac death; SCD, sudden cardiac death*.

* *Adjusted for: age, sex, BMI, NYHA class, ejection fraction, ischemic etiology, non-sustained ventricular arrhythmias, use of beta-blocker*.

**Figure 3:**
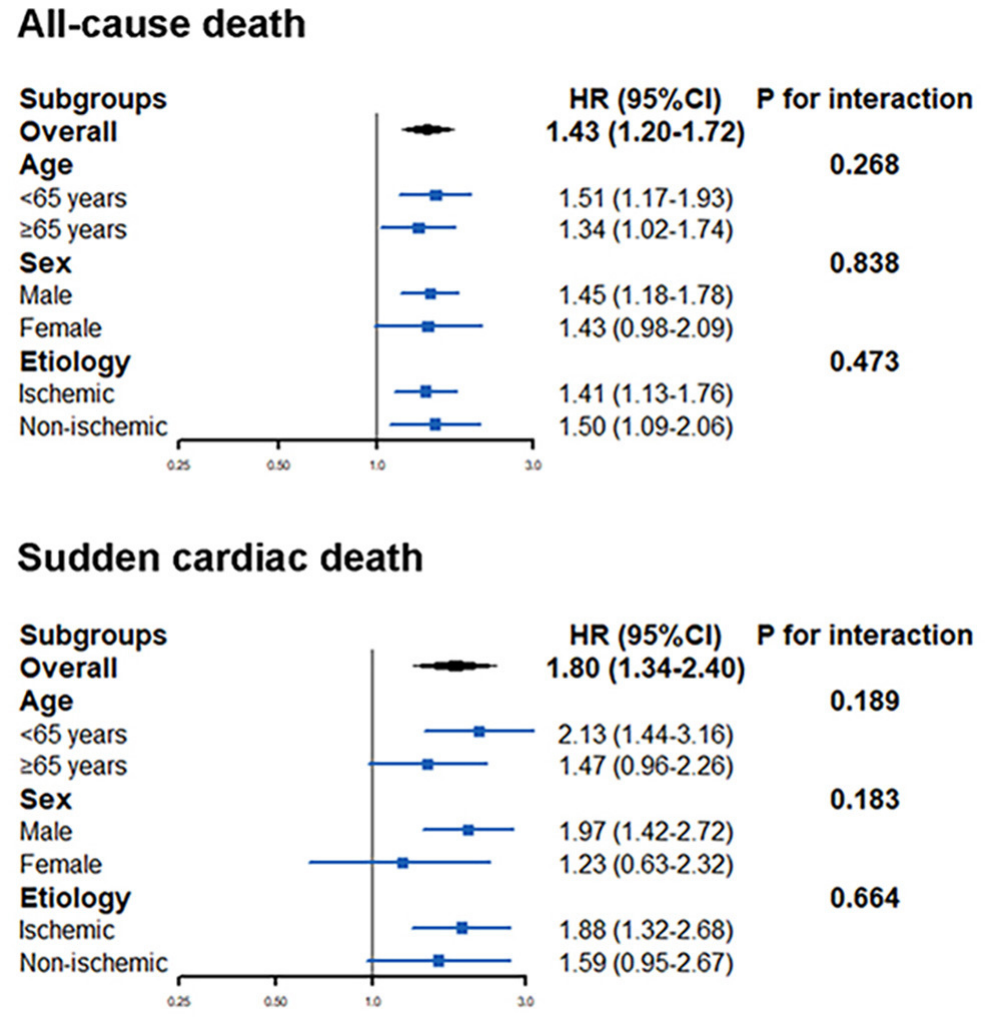
The effect of A allele of rs2302684 on all-cause mortality and sudden cardiac death in different subgroups, including different ages, sex, and etiology.

The effect of A allele of rs2302684 on the mortality endpoints was generally consistent across the selected subgroups, including different age (*P* for interaction = 0.268 for all-cause mortality and 0.189 for SCD), sex (*P* for interaction = 0.838 for all-cause mortality and 0.183 for SCD), and ischemic etiology (*P* for interaction = 0.473 for all-cause mortality and 0. 664 for SCD) (Figure 3).

## Discussion

### Main Findings

In this prospective study of 1,437 patients with CHF in the Chinese Han nationality, the associations of SNP rs2302684 T>A in *LRP6* and long-term clinical endpoints were explored. The A allele of rs2302684 was recognized as an independent risk factor and predictor of all-cause death and SCD in the CHF population. To the best of our knowledge, this is the first study to show the association between common variants in *LRP6* gene with the different causes of mortality in patients with CHF.

### *LRP6* and Wnt Signal Regulation

The human *LRP6* gene, which is located in chromosome 12 p11–p13, has 150 kb in length with 23 exons (8, 19). It encodes a member of the low-density lipoprotein receptor family, which is composed of cell surface proteins that participate in receptor-mediated endocytosis of specific ligands (8, 19). *LRP6* gene is homologous to *LRP5* and has large extracellular domains consisting of four β-propeller motifs followed by three LDL ligand-binding domains, which regulate the binding process between Wnt secretory protein and frizzled family of receptors (10, 20). LRP6 function as coreceptors for Wnt ligands and thus play a central role in Wnt/β-catenin signaling involved in a wide variety of biologic processes (12, 21).

Wnts are a family of secreted glycoproteins that participate in activating several signaling pathways (22). It could bind to a class of Frizzled receptors or LRP6 to downregulate the glycogen synthase kinase-3β (GSK-3β) activity and to initiate the canonical Wnt/β-catenin signaling cascade (22). Wnt/β-catenin signaling is a crucial regulator of tissue development and homeostasis, especially in cardiac differentiation and development (11, 20). The conserved Wnt cascade has been confirmed to control the proliferation, differentiation and polarity of cells (23–25). Abnormal signaling disturbs tissue growth and function, which could lead to a number of debilitating and terminal diseases (20). Thus, alterations in the *LRP6* gene might affect Wnt/β-catenin signaling and lead to several human diseases including osteoporosis, Alzheimer's disease, coronary artery disease, and metabolic disease (20, 26–31).

### *LRP6* and Sudden Cardiac Death

Wnt signaling is critical in cardiac development and various cardiac pathologies, including cardiac hypertrophy and fibrosis, myocardial infarction, heart failure, and arrhythmias (30, 32, 33). It has been reported that abnormalities of Wnt signaling were an important cause of familial sudden death in patients with ARVC (34). Additionally, a recent genome-wide association study showed that WNT8A was associated with atrial fibrillation (35).

Mutations in *LRP6* could dysregulate Wnt signaling and have been associated with numerous human diseases (12, 20). The rs2302684 is an intron variant. No association has been reported between this tag SNP of *LRP6* and the prognosis of patients with CHF in the Chinese Han population. Our study revealed a close relationship between *LRP6* common variants and SCD in the CHF group. According to previous researches, the potential reasons for this relationship might have two aspects. On one hand, LRP6 serves as a scaffold protein that regulates the cardiac gap junction assembly. *LRP6* deficiency might impair the dynamics of connexin43 protein trafficking and stability, which disrupts gap junction formation and function. The proper functioning of gap junctions is essential in the generation and propagation of cardiac action potentials. Thus, the disrupting connexin43 expression or phosphorylation caused by *LRP6* deficiency impaired the electrical communication in gap junctions and led to the initiation and maintenance of arrhythmias (33, 36, 37). It was reported that defective connexin43 gap junctions in *LRP6*-ablated mouse hearts induced VT and VF (13). On the other hand, many studies demonstrated that Wnt signaling was linked to cardiac fibrosis which could impede electrical wave propagation and potentially cause arrhythmias (38–40). Furthermore, a recent study reported that *LRP6* played an important role in keeping the integrity of the intercalated disk, on which the coordinated excitation and contractile performance of the myocardium were dependent, and the interaction between *LRP6* and connexin43 might be involved in this process (41). Therefore, *LRP6* variants might cause malignant arrhythmias and SCD via disturbing Wnt signaling pathways, as well as disrupting the function of gap junction and the intercalated disk of the myocardium.

### *LRP6* and CHF With Different Etiologies

It was reported that LRP6 was dramatically decreased in heart tissues with DCM (42). Additionally, *LRP6* is genetically linked to early coronary artery disease and hyperlipemia (20, 43, 44). It has been demonstrated that mutant *LRP6* was associated with atherosclerosis. The underlying mechanism might be as follows: firstly, LRP6 is critical in LDL receptor-mediated LDL uptake, which is significant in atherosclerosis; secondly, LRP6 plays an important role in metabolic regulation, including lipid homeostasis and glucose metabolism, thus it is associated with atherosclerosis; lastly, mutant *LRP6* could trigger atherosclerosis by activating platelet-derived growth factor (PDGF)-dependent vascular smooth muscle cell differentiation (31, 45). Considering the close relationship of *LRP6*, DCM, and atherosclerosis, we analyzed the association of *LRP6* with the mortality endpoints in different etiologies of CHF. In our study, the effect of rs2302684 A allele in *LRP6* on the mortality endpoint was consistent in patients with different CHF reasons, including ICM and DCM. Therefore, we found that *LRP6* variants were associated with a higher risk of all-cause death and SCD in the CHF cohort attributed to both ICM and DCM.

### Study Limitations

There are several limitations to the study. The lack of functional research in this work is one of the limitations. In this study, we only found the association between the SNP of *LRP6* and SCD in CHF patients via gene tests, however, the exact mechanism of how the SNP affects the heart is still unknown. We only speculate the possible mechanism according to existing studies, and we still need further functional studies to explore the underlying mechanism in our future work. Additionally, ICD recordings were not collected in the present cohort, and only a limited number of patients had a history of ventricular arrhythmias (VAs). Therefore, we are not able to deepen our investigation about the association between VAs and the SNP of interest.

## Conclusions

The study firstly demonstrated that *LRP6* rs2302684 polymorphism is associated with increased risks of all-cause death and SCD in CHF patients in the Chinese Han population. Therefore, *LRP6* could be regarded as an independent risk factor and a novel predictor of SCD, and it might provide a potential therapeutic target in SCD prevention.

## Data Availability Statement

The datasets presented in this article are not readily available because the hospital does not permit the authors to publicize the database. Requests to access the datasets should be directed to the corresponding author/s.

## Ethics Statement

The studies involving human participants were reviewed and approved by Anzhen Hospital and Fuwai Hospital. The patients/participants provided their written informed consent to participate in this study.

## Author Contributions

QG, YL, JP, and CM is responsible for the conception and design of the study. QG conducted the selection of the SNP. QG and MG conducted the telephone interview. JC and XC conducted the in-person interview. QG and YL was responsible for the statistical analysis and drafted the manuscript. JD and CS revised it critically for important intellectual content. JP and CM critically appraised the manuscript and approved the final version. All authors have the access to all of the data, read the manuscript, and agreed to be accountable for all aspects of the work.

## Funding

This work was supported by the National Key Research and Development Program of China (2016YFC0900901) to CM and the National Basic Research Program of China (2013CB531105) to JP.

## Conflict of Interest

The authors declare that the research was conducted in the absence of any commercial or financial relationships that could be construed as a potential conflict of interest.

## Publisher's Note

All claims expressed in this article are solely those of the authors and do not necessarily represent those of their affiliated organizations, or those of the publisher, the editors and the reviewers. Any product that may be evaluated in this article, or claim that may be made by its manufacturer, is not guaranteed or endorsed by the publisher.

## Abbreviations

CHF: chronic heart failure
DCM: dilated cardiomyopathy
ICM: ischemic cardiomyopathy
LRP6: low-density lipoprotein receptor-related protein 6
LVEF: left ventricular ejection fraction
PCR: polymerase chain reactions
SCD: sudden cardiac death.
